# Exploring the lutein therapeutic potential in steatotic liver disease: mechanistic insights and future directions

**DOI:** 10.3389/fphar.2024.1406784

**Published:** 2024-06-24

**Authors:** Elisa Balboa, Faride Saud, Claudia Parra-Ruiz, Marjorie de la Fuente, Glauben Landskron, Silvana Zanlungo

**Affiliations:** ^1^ Center for Biomedical Research, Universidad Finis Terrae, Santiago, Chile; ^2^ Department of Gastroenterology, Faculty of Medicine, Pontificia Universidad Católica de Chile, Santiago, Chile

**Keywords:** hepatic steatosis, lipophagy, lutein, TFEB, StARD3, lipid droplet

## Abstract

The global prevalence of Metabolic Dysfunction-Associated Steatotic Liver Disease (MASLD) is increasing, now affecting 25%–30% of the population worldwide. MASLD, characterized by hepatic steatosis, results from an imbalance in lipid metabolism, leading to oxidative stress, lipoperoxidation, and inflammation. The activation of autophagy, particularly lipophagy, alleviates hepatic steatosis by regulating intracellular lipid levels. Lutein, a carotenoid with antioxidant and anti-inflammatory properties, protects against liver damage, and individuals who consume high amounts of lutein have a lower risk of developing MASLD. Evidence suggests that lutein could modulate autophagy-related signaling pathways, such as the transcription factor EB (TFEB). TFEB plays a crucial role in regulating lipid homeostasis by linking autophagy to energy metabolism at the transcriptional level, making TFEB a potential target against MASLD. STARD3, a transmembrane protein that binds and transports cholesterol and sphingosine from lysosomes to the endoplasmic reticulum and mitochondria, has been shown to transport and bind lutein with high affinity. This protein may play a crucial role in the uptake and transport of lutein in the liver, contributing to the decrease in hepatic steatosis and the regulation of oxidative stress and inflammation. This review summarizes current knowledge on the role of lutein in lipophagy, the pathways it is involved in, its relationship with STARD3, and its potential as a pharmacological strategy to treat hepatic steatosis.

## 1 Introduction

Metabolic dysfunction-associated steatotic liver disease (MASLD) poses a significant challenge to public health, impacting a substantial proportion of the population, with approximately 30% of individuals affected globally. Particularly concerning is its escalating prevalence in Latin America (Arab et al., 2021). Despite its growing significance, there remains a notable absence of targeted pharmacological interventions for this condition.

It is well established that a carotenoid-rich diet benefits liver health (Elvira-Torales et al., 2019). Dietary carotenoids are mainly accumulated in the liver, where they are packaged into different lipoproteins for their release to the blood circulation and other organs and tissues, such as the kidneys, adipose tissue, skin, brain, and retina (Elvira-Torales et al., 2019). The accumulation of these antioxidants and their metabolites in the liver allows to rise carotenoids as a promising therapy for liver diseases. Individuals who consume the highest amounts of carotenoids have the lowest risk of developing MASLD (Ferramosca et al., 2017).

Among the array of carotenoids garnering attention for their hepatoprotective properties, lutein stands out for its antioxidant, anti-inflammatory, and hypolipidemic effects. (Cao et al., 2015). Lutein supplementation in rats fed with a high-fat diet (HFD), a model of MASLD, recovered liver function, improved lipid accumulation, and restored hepatic lipid metabolism and insulin signaling, preventing hepatic dyslipidemia and insulin resistance (Wang et al., 2021). In addition, lutein reduced lipid peroxidation and decreased pro-inflammatory cytokine production in the liver of guinea pigs fed with a high-cholesterol diet (Kim et al., 2012). Moreover, in rats with hepatic injury, lutein lowers ALT and AST concentrations, two markers of liver damage (Sindhu et al., 2010). In summary, there is mounting evidence that lutein has beneficial effects on the liver. At the same time, its anti-oxidative potential has been well-documented, but the precise mechanisms underlying its hypolipidemic effects remain inadequately explored. This review aims to consolidate existing evidence and elucidate the potential mechanism by which lutein may exert its hypolipidemic effects, possibly through the modulation of autophagy via the activation of the master transcription factor TFEB.

TFEB is a crucial regulator of cellular processes, among them lysosomal biogenesis and autophagy, and its signaling pathways influence cellular energy metabolism. Its activity and localization are modulated by mTORC1-mediated phosphorylation, determining its subcellular localization in the cytoplasm or nucleus, thus its function as a transcription factor. TFEB’s involvement includes lipid catabolism and it’s a clear potential therapeutic target mediating cellular clearance in a variety of diseases, including rare lysosomal storage diseases and more prevalent ones such as Parkinson’s and Alzheimer’s (Napolitano and Ballabio, 2016).

Furthermore, we propose a putative intracellular transport pathway for lutein mediated by STARD3, drawing upon insights from studies on the intracellular trafficking of other carotenoids. STARD3 is a cholesterol and lutein-binding protein found in late endosomes, featuring a domain that anchors it to endosome membranes and facilitates interactions with other proteins. Its exact role remains unclear, but it’s known to create contact sites between the endoplasmic reticulum (ER) and endosomes potentially influencing cholesterol and lipid distribution within cells (Alpy et al., 2013).

This investigation holds substantial importance as it offers prospects for therapeutic advancements in hepatic steatosis and promises insights into its underlying pathophysiology. This article delineates the current understanding of lutein’s mechanisms and its therapeutic potential in addressing fatty liver disease.

## 2 Metabolic dysfunction-associated steatotic liver disease

Between 25 and 30 percent of the general population suffers from Metabolic dysfunction-associated steatotic liver disease (MASLD), formerly known as non-alcoholic fatty liver disease or NAFLD. It has become the most frequent cause of liver disease worldwide (Asrani et al., 2019). Higher prevalence rates are found in particular subpopulations, such as people with obesity (90%) and type 2 diabetes (76%) (Younossi et al., 2016). Furthermore, metabolic disorders frequently coexist with the onset of MASLD syndrome, including increased triglycerides (TGs, neutral fats) in the plasma, insulin resistance, hypertension, and reduced high-density lipoprotein (HDL) cholesterol. Hepatic steatosis, defined as the intracytoplasmic TGs accumulation, representing at least 5% of liver weight, is the defining characteristic of MASLD (Chalasani et al., 2018). Most patients have simple steatosis, but steatohepatitis (liver inflammation) is present in about 25% of MASLD patients. Although this inflammatory injury is reversible, it can progress to an irreversible stage of liver damage such as fibrosis, cirrhosis, and hepatocellular carcinoma (Haas et al., 2016), being crucial to intervene therapeutically at the reversible stages of the disease.

Hepatic free fatty acids (FFAs) can be acquired from the diet, adipose tissue lipolysis, and *de novo* lipogenesis. FFAs are then oxidized through β-oxidation or esterified into TGs, stored as lipid droplets (LD), or packaged into lipoproteins, such as VLDL, to be secreted into the bloodstream (Nassir et al., 2015). The imbalance among these major molecular pathways and how their dysregulation contributes to the development of MASLD is not the focus of discussion in this review. However, an important pathway that may contribute to MASLD is lipolysis, which will be discussed below.

### 2.1 Lipolysis

Lipolysis is the process by which lipase hydrolyzes LDs, releasing FFAs and glycerol (Ducharme and Bickel, 2008; Grabner et al., 2021). This metabolic process occurs in several tissues throughout the body, including adipose tissue, liver, muscle, and heart. In adipose tissue, lipolysis primarily aims to release FFAs and glycerol for energy production. The rate-limiting step in adipocyte TG lipolysis is LD-associated adipose triglyceride lipase (ATGL). ATGL generates diacylglycerol (DG), which is further hydrolyzed by hormone-sensitive lipase (HSL) to release monoglycerides (MG). This process is tightly regulated by hormones such as adrenaline and glucagon, which activate HSL (Grabner et al., 2021). Conversely, hepatic lipolysis is vital for lipid metabolism and lipoprotein production. It is controlled through the action of the ATGL and is influenced by factors such as insulin, glucagon, and FFA levels (Alves-Bezerra and Cohen, 2017). Hepatic lipolysis contributes to the synthesis of VLDL, which plays a crucial role in maintaining lipid homeostasis and distributing lipids throughout the body. Additionally, during extended periods of fasting or in low-carbohydrate conditions, hepatic lipolysis becomes pivotal as the liver mobilizes stored fatty acids through this process to produce acetyl-CoA for utilization in beta-oxidation for ATP production. This acetyl-CoA is an alternative energy source for various tissues, notably the brain, when blood glucose levels are low (Mohiuddin., 2023).

The LD components also regulate LD lipolysis. Phospholipids and proteins surround LD cores. LD-associated proteins, including perilipins (PLIN), are crucial in LD formation and degradation. PLIN2 and PLIN3 are associated with LDs in hepatocytes, and their phosphorylation and degradation are necessary for lipases to access the LD during lipolysis (Alves-Bezerra and Cohen, 2017).

Intracellular lipases can be categorized into two subclasses: neutral lipases, which degrade cytosolic LDs, and acid lipases, which are localized at lysosomes. Consequently, the two main intracellular pathways responsible for TG degradation are referred to as neutral and acid lipolysis.

In neutral lipolysis, TGs undergo consecutive hydrolysis by ATGL, HSL, and MGL (monoacylglycerol lipase), producing FFAs and glycerol. On the other hand, acid lipolysis involves the sequestration of TGs from LDs through the autophagy of lipids, also known as lipophagy, followed by hydrolysis by lysosomal acid lipase (LAL) within lysosomes, generating FFAs and glycerol (Onal et al., 2017).

Lipophagy is a relatively recent discovery compared to the pathways described above. Moreover, disturbances in lipophagy have been linked to MASLD and hepatic TG accumulation (Grefhorst et al., 2020; Feng et al., 2023).

### 2.2 Lipophagy in MASLD

Lipophagy is a novel form of lipid catabolism that involves the autophagic degradation of intracellular lipid droplets. Autophagy is a tightly regulated process, and the key players in this pathway are the autophagy-related (ATG) proteins. Autophagy impairment has been strongly associated with MASLD pathogenesis. Accordingly, hepatocytes exposed to control and FFAs, treated with autophagy inhibitors or (sh)RNA against the autophagy gene *Atg5*, which encodes the protein ATG5 essential for autophagic vesicle formation, showed increased lipid droplet content. In addition, mouse embryonic fibroblasts knocked out for *Atg5* also displayed increased triglyceride levels (Singh et al., 2009).

Moreover, liver-specific deletion of *Atg7*, whose protein stimulates phagophore expansion, increases hepatic fat content in mice, mimicking the human condition of hepatic steatosis (Martinez-Lopez and Singh, 2015). Interestingly, in obesity models, such as high-fat-diet-fed mice and the leptin-deficient *ob/ob* mice, it has been reported that chronic overnutrition decreases hepatic ATG7 levels and autophagy activity, being liver-specific ATG7 overexpression sufficient to prevent hepatic steatosis (Yang et al., 2010). Furthermore, overnutrition-induced autophagy suppression has been associated with hepatic inflammation and ER stress (Martinez-Lopez and Singh, 2015), supporting the central role of autophagy in controlling hepatic fat content and in the pathogenesis of MASLD.

Essential signaling pathways regulate autophagy in the cell, including stress-signaling kinases such as JNK-1, which promotes autophagy by phosphorylating BCL-2, thereby enabling the interaction of Beclin-1 with VPS34 (Wei et al., 2008). The central signaling molecule that determines the levels of autophagy in cells is the mammalian target of rapamycin (mTOR) kinase. In cells, mTOR manifests as two functionally and structurally distinct complexes known as mTOR complex 1 (MTORC1) and mTOR complex 2 (MTORC2). MTORC1 serves as a transcription-independent regulator of autophagy. In conditions of nutrient availability, MTORC1 becomes active and inhibits, by direct phosphorylation, ATG proteins involved in autophagy induction, such as ATG13 and ATG1 (ULK1/2). Conversely, during periods of nutrient deprivation, MTORC1 deactivation prompts its dissociation from the ULK complex, leading to autophagy induction (Rabanal-Ruiz et al., 2017).

An important mechanism governing autophagy through transcription has been described, involving the transcription factor EB (TFEB). TFEB belongs to the basic helix-loop-helix leucine-zipper family of transcription factors. It governs lysosomal biogenesis and autophagy by positively modulating genes within the Coordinated Lysosomal Expression and Regulation (CLEAR) network (Zhu et al., 2021).

TFEB undergoes intricate regulation through phosphorylation and dephosphorylation processes. Phosphorylation of crucial serine residues, such as S122, S142, and S211, by mTORC1, inhibits TFEB’s activity, sequestering it in the cytoplasm during nutritional sufficiency (Settembre and Ballabio, 2011). Multiple serine kinases, including AKT, GSK3β, ERK2, and MAPK4, can phosphorylate TFEB (Zhu et al., 2021) as well as tyrosine kinase c-Abl (Contreras et al., 2020). Conversely, under nutrient deprivation conditions, calcineurin plays a pivotal role in dephosphorylating and activating TFEB (Medina et al., 2015). Notably, calcineurin’s effects on TFEB activation override those of mTORC1, indicating a downstream or parallel regulatory pathway (Medina et al., 2015). Furthermore, TFEB regulation can be influenced by ER stress and reactive oxygen species (ROS), which may directly or indirectly enhance calcineurin’s impact on TFEB through the lysosomal calcium channel, mucolipin 1 (MCOLN1) (Martina et al., 2016). This multifaceted regulatory network governs TFEB’s subcellular localization and activity, aligning it with nutrient availability and particular cellular conditions.

### 2.3 TFEB in hepatic lipid metabolism

TFEB exerts a central role in hepatic lipid metabolism by orchestrating a transcriptional program in response to starvation via the PGC1α-PPARα-lipophagy axis that mediates lipid catabolism. Moreover, evidence shows that the cAMP response element-binding protein (CREB) promotes lipophagy in the fasted state via direct transcriptional activation of TFEB and autophagy genes (Seok et al., 2014).

Previously, it has been shown that TFEB overexpression/activation induces an increased number of autophagosomes and autophagic flux, generation of new lysosomes, and leads to clearance of storage material in lysosomal storage disorders (LSDs) (Contreras et al., 2020; Arguello et al., 2021). Notably, human livers with steatosis or NASH exhibit impaired autophagy with reduced nuclear TFEB (Kim et al., 2017a). Moreover, pharmacological TFEB activation improves hepatic lipid accumulation and β-oxidation in diet-induced NAFLD animal models (Sinha et al., 2014; Zhou et al., 2014). Ezetimibe, a drug that decreases plasma cholesterol levels, ameliorates steatohepatitis through AMPK activation and TFEB nuclear translocation, thereby inducing autophagy (Kim et al., 2017a). Expanding on these findings, a study on hepatocytes treated with palmitic acid, an *in vitro* fatty liver disease model, revealed a decrease in nuclear TFEB levels and its downstream target genes encoding for lysosomal CTSB and LAMP1. However, this reduction was counteracted by liraglutide treatment, a glucagon-like peptide-1 receptor agonist (GLP-1RA) known for its steatosis-reducing effects. Crucially, the beneficial impact of liraglutide on steatosis was hindered by TFEB downregulation (Fang et al., 2020). Consistent with these observations, the active alkaloid ajugol, derived from Rehmannia glutinosa root, effectively alleviated high-fat diet-induced hepatic steatosis in mice and inhibited palmitate-induced lipid accumulation in hepatocytes through TFEB’s nuclear translocation (Zhang et al., 2021). Moreover, Philadelphia genin (PHI), a lignin derived from Forsythia suspense, demonstrates significant hepatoprotective and anti-inflammatory effects both *in vitro* and *in vivo* (high-fat diet (HFD) mice). PHI functions by enhancing lysosomal biogenesis and autophagic flux, thereby reducing lipid accumulation. Additionally, PHI inhibits the NLRP3 inflammasome pathway, mitigating hepatocyte inflammation in an autophagy-TFEB-dependent manner, as evidenced by the abolition of these effects upon TFEB inhibition (Zhou et al., 2022). Interestingly, it has been observed that genistein, a TFEB activator (Arguello et al., 2021), has beneficial effects on NAFLD (Xin et al., 2019), once again suggesting that TFEB activation helps treat the disease. These results in the mentioned steatosis models position TFEB as a promising therapeutic target for the disease and warrant the rational search for compounds capable of activating TFEB and reducing hepatic steatosis.

Additionally, TFEB dysregulation has been implicated in various other liver pathologies (Yan, 2022), positioning it as a viable treatment strategy not only for metabolic-associated fatty liver disease (MAFLD) but also for alcohol-induced steatosis (AIS) and other hepatic disorders.

## 3 Lutein

Lutein is one of the most abundant carotenoids in human blood samples, together with lycopene, β-carotene, β-cryptoxanthin, α-carotene, and zeaxanthin (Torsten, 2008). It is a non-provitamin A carotenoid that belongs to the xanthophyll family and is found in spinach, kale, eggs, corn, and green leafy vegetables (Abdel-Aal el et al., 2013). It is known to accumulate in human and primate retina and brain selectively (Erdman et al., 2015), being one of two major carotenoids found as a color pigment in the human eye (macula and retina). It functions as a light filter, protecting the eye tissues from sunlight and oxidative damage (Buscemi et al., 2018). Recently, lutein has drawn increasing attention to its function in chronic diseases other than oculopathy. It has been reported that lutein has anti-inflammatory, antiapoptotic, and anti-oxidative properties through the blockage of the NF-κB pathway (Li et al., 2012a; Li et al., 2012b) and by decreasing IL-6 (Ozawa et al., 2012; Ahn et al., 2022), among other pathways in different pathological conditions, including atherosclerosis and cerebral ischemia/reperfusion models. Lutein is also being studied as a neuroprotective agent (Sun et al., 2014) and as a cognitive performance enhancer (Johnson, 2012); Clinical trials have supported the beneficial effect of oral supplementation with 10 mg of lutein in Alzheimer’s disease patients, particularly in terms of clinically meaningful improvements in visual function but not in cognitive function (Nolan et al., 2015). Furthermore, ongoing clinical trials are evaluating the effect of lutein in reducing oxidative stress caused by amyloid beta (Aβ) in Alzheimer’s patients, although the results of these trials have not yet been published^1^. Also in rats Lutein decreased the Aβ effects on learning and memory (Nazari et al., 2022). However, its role in liver pathologies has been little researched, but the evidence suggests that lutein would be a promising therapy for liver disease.

Lutein supplementation in rats increased the expression of critical factors in autophagy signaling, such as phosphatidylinositol 3-kinase, an upstream regulator of TFEB (Qiu et al., 2015). Also, a high dose of lutein increased the expression of PPARα (Qiu et al., 2015), which is upstream of TFEB (Kim et al., 2017b) and is associated with lipid metabolism, as previously described (Ghosh et al., 2015). Also, long-term oral feeding of lutein increases total AMP-activated protein kinase (tAMPK) and phosphorylated AMP-activated protein kinase (pAMPK) contents in muscle (Matsumoto et al., 2014), and there is abundant evidence linking AMPK phosphorylation with TFEB activation (Collodet et al., 2019; Huang et al., 2019; Paquette et al., 2021). Moreover, in a model of rat intestinal epithelial cells, lutein treatment induces the processing of LC3-II and the upregulation of autophagy-related genes, including *Atg4A, Atg5, Atg7, Atg12,* and *Becn1* (Chang et al., 2017). On the other hand, there is evidence that lutein inhibits autophagy in rat müller cells exposed to hypoxia (Fung et al., 2016), suggesting that the lutein effect on autophagy could depend on the cellular type. This compelling evidence suggests that lutein may contribute to the induction of autophagy, mediated by the activation of TFEB, PI3K/Akt, and AMPK pathways. However, there is a lack of relevant studies in this area, warranting further investigation to conclusively establish the role of lutein in autophagy.

### 3.1 Hepatoprotective effects of lutein

Lutein supplementation in rats fed with a high-fat diet (HFD), a model of MASLD, recovered liver function, improved lipid accumulation, and restored hepatic lipid metabolism and insulin signaling through regulation of the protein Sirtuin 1(SIRT1), suggesting that lutein supplementation plays a potential role in preventing hepatic dyslipidemia and insulin resistance (Qiu et al., 2015). Lutein reduced lipid peroxidation and decreased pro-inflammatory cytokines TNF-α, NF-κβ and IL-1β production in the liver and eye of guinea pigs fed with high cholesterol diet (Kim et al., 2012), supporting the role of lutein as a hepatoprotective molecule. Moreover, in rats with hepatic injury induced by paracetamol, carbon tetrachloride, and ethanol, lutein lowers ALT and AST concentrations, two markers of liver damage (Sindhu et al., 2010). In alcohol-induced liver injury, lutein decreases serum liver markers, enhances the antioxidant status, and exerts anti-inflammatory effects (Du et al., 2015; Zhao et al., 2023).

### 3.2 Lutein absorption and metabolism

Lutein is not synthesized in mammals; it must be obtained from the diet. During digestion, carotenoids and fat-soluble vitamins are incorporated with other lipids into the mixed micelles (Borel, 2003). In the enterocyte, lutein is captured from mixed micelles by the apical membrane transporters: scavenger receptor class B type I (SR-BI) (Reboul et al., 2005), cluster determinant 36 (CD36) (Moussa et al., 2011) and Niemann–Pick C1-Like 1 (NPC1L1) (Sato et al., 2012).

The contribution of SR-BI to intestinal lutein transport was analyzed using Caco-2 monolayers as a model for human intestinal epithelium. In these cells, the treatment with anti-SR-BI antibody significantly impaired the lutein absorption rate by approximately 30%. Also, the treatment with BLT1, a chemical inhibitor of the selective transfer of lipids mediated by SR-BI, reduced lutein absorption by roughly 57%, indicating that some lutein is taken up through SR-BI (Reboul et al., 2005). Also, using Caco-2 monolayers, it was determined that NPC1L1 participates in the transport of lutein in the enterocyte since the treatment with ezetimibe, an NPC1L1 selective inhibitor, decreased 40% of the lutein transport (Sato et al., 2012).

It has been shown that CD36 is involved in the transport of lutein in other cell types, such as adipocytes. However, different proteins are probably involved in its uptake since specifically inhibiting CD36 does not totally decrease lutein transport (Moussa et al., 2011).

**FIGURE 1:**
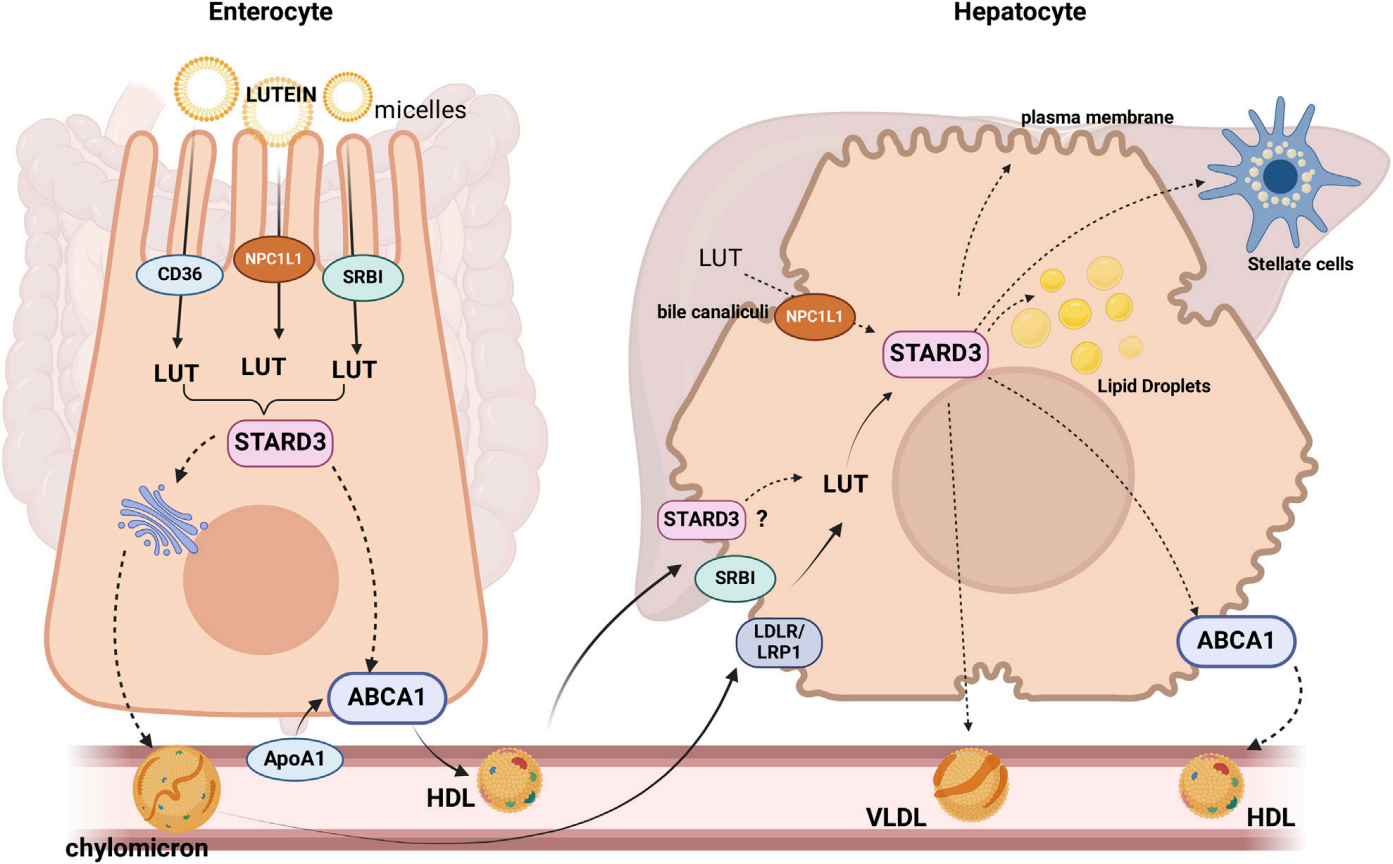
Proteins or candidate proteins involved in lutein (LUT) absorption, uptake, and trafficking in enterocytes and hepatocytes: After being hydrolyzed, esterified lutein (LUT) can be taken up by the enterocyte through the receptors, SR-B1, CD36 and NPC1L1 (Sato et al., 2012). There are two different pathways by which lutein may efflux from the enterocytes, one of which is the assembly into chylomicrons (QM) and release into the lymph, or it can be secreted directly into high-density lipoprotein (HDL) (Murillo et al., 2019). STARD3 would mediate the intracellular transport of lutein. STARD3, SRBI, and LDLR/LRP1 would participate in the lutein uptake by the liver, where it can be stored, eliminated in the bile, or re-distributed to extrahepatic tissues via lipoprotein secretion. SRBI: scavenger receptor class B type 1. NPC1L1: Niemann-Pick C1-Like 1. The dotted lines indicate proposed mechanisms.

Much less is known about the intracellular transport of lutein. We propose that selective lutein-binding proteins may be involved. Steroidogenic Acute Regulatory (StAR)-related lipid transfer domain–3 (STARD3) is a good candidate for mediating lutein intracellular transport. This protein contains a StAR-related lipid transfer (START) domain, which serves as the lipid and lutein binding site (Li et al., 2011). STARD3 is expressed in enterocytes (Ross et al., 2017), and it has been proposed that it may facilitate the delivery of lutein at the basolateral side via ABCA1 (apoAI pathway) to form HDL (Reboul, 2013) or to the ER for its incorporation into chylomicrons (Figure 1). After that, lutein exits from the enterocyte and enters the circulation as a component of HDL and chylomicrons.

**FIGURE 2:**
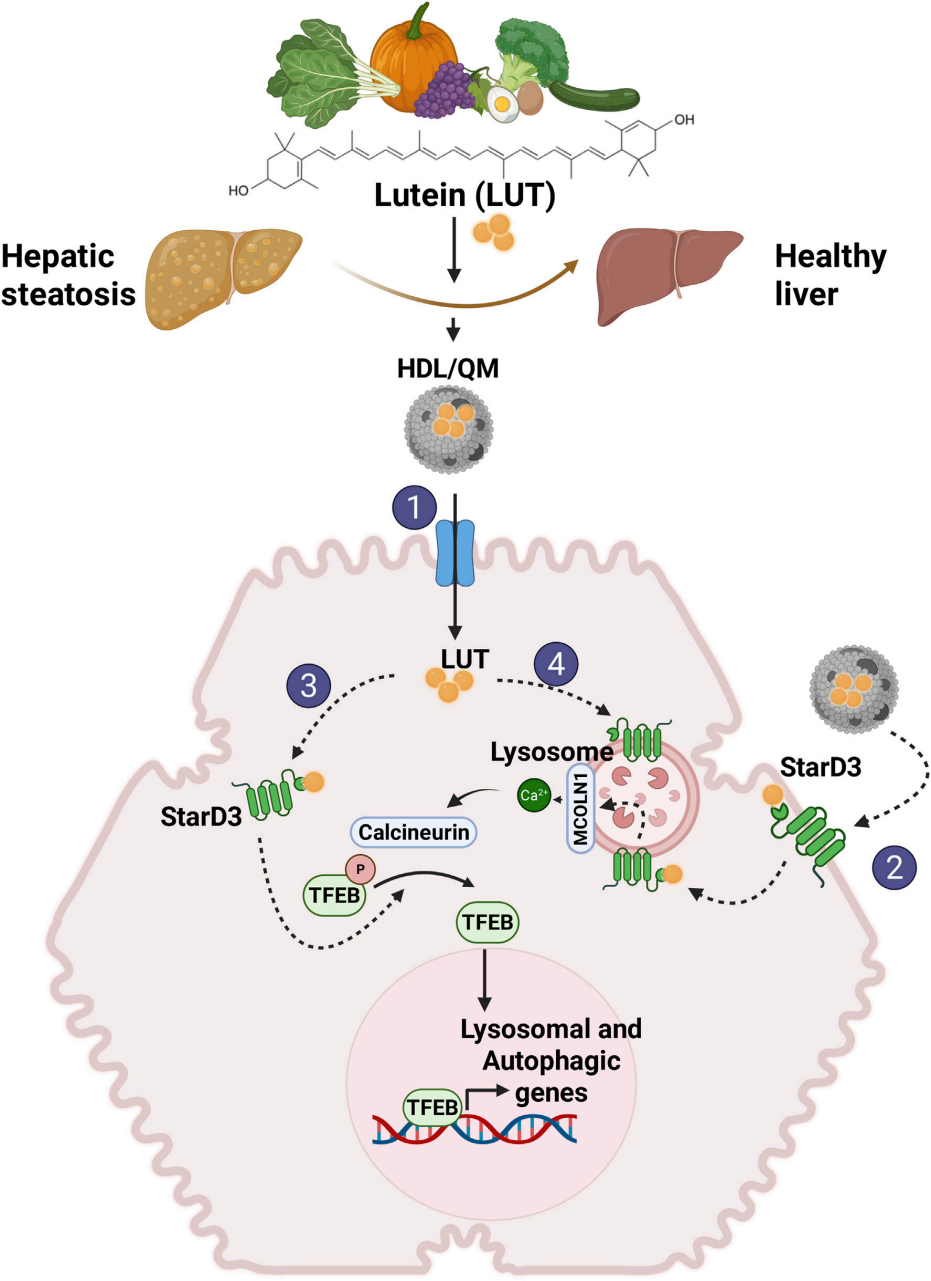
Proposed mechanisms involved in lutein-mediated hepatoprotective effects. We propose that independent of its antioxidant and antiapoptotic activity, lutein stimulates LD autophagy in the liver, reducing their accumulation and improving steatosis. This action is mediated through the activation of TFEB. Given lutein’s hydrophobic nature as a carotenoid, an intracellular transporter is required to exert its activity within the cell. We propose that STARD3 serves as this transporter. 1) To enter the cell, lutein in lipoproteins, primarily QM and HDL, binds to their transporters on the hepatocyte membrane (SRBI, LDLR, LRP1). 2) Additionally, lutein may directly enter the cell by binding to STARD3 on the plasma membrane. 3) Once inside the cell, lutein bound to STARD3 would directly or indirectly interact with TFEB, mediating its activation. 4) Another pathway for lutein entry involves endosomal uptake, where it binds to STARD3 in the lysosome, activating TFEB via the MCOLN1/Ca^2+^/calcineurina pathway.

Lutein uptake by the liver has not been characterized, but retinol transport, another carotenoid, is fully described. In the liver, retinol enters the hepatocyte via receptor-mediated endocytosis mainly by the LDL receptor (LDLR), then is rapidly hydrolyzed, bound to retinol-binding protein (RBP), re-esterified and stored as retinyl esters within cytoplasmic LD or destined to the hepatic stellate cells (Ross et al., 2017). Based on the evidence discussed above, it is reasonable to hypothesize that lutein could enter the hepatocyte using chylomicrons and HDL via receptor-mediated endocytosis through chylomicron remnant receptors (LRP1, LDLR) or selective uptake mediated by SRBI, respectively (Rigotti et al., 2003). In the cytoplasm, lutein could be transported by STARD3, the only protein probed to bind lutein (Li et al., 2011), and finally, transferred to the lipid bilayer or re-packaged into lipoproteins (Figure 2). In the liver, lutein is incorporated into HDL (approx. 52%) and, to a lesser extent, into LDL (22%) and VLDL (Wang et al., 2007), which deliver lutein to target tissues.

## 4 The lutein-binding protein STARD3

Steroidogenic Acute Regulatory (StAR)-related lipid transfer Domain–3 (STARD3), also known as Metastatic axillary Lymph Node clone 64 (MLN64) (Tomasetto et al., 1995), is a late endosome (LE) membrane-associated protein (Alpy et al., 2001), which is evolutionarily conserved throughout the animal kingdom, as well as in unicellular organisms closely related to animals (Voilquin et al., 2019). STARD3 belongs to the START family of proteins, whose members share a conserved StAR-related lipid transfer (START) domain. The START family comprises 15 members, with STARD3 being the only member with a transmembrane anchoring domain (Clark, 2020; Asif et al., 2021).

START proteins have a conserved fold, but their primary sequences differ, allowing selectivity in ligand binding. These proteins are involved in the transport of lipids such as sterols, phospholipids, bile acids, ceramides, and carotenoids (Alpy and Tomasetto, 2014; Clark, 2020; Sluchanko et al., 2022).

STARD3 has been identified as the unique protein with high-affinity binding to lutein (Li et al., 2011). Primarily recognized for its role in cholesterol binding, STARD3 serves as a transporter facilitating cholesterol transfer between the late-endosome/lysosome (LE/Lys) and the endoplasmic reticulum (ER) by forming Membrane Contact Sites (MCSs) (Alpy et al., 2001; Alpy et al., 2013; Wilhelm et al., 2017). Functionally, it has been implicated in cholesterol transport during steroidogenesis in placental tissue (Watari et al., 1997; Bose et al., 2000; Zhang et al., 2002). Despite the established involvement of STARD3 in cholesterol mobilization towards the mitochondria (Hoglinger et al., 2019; Nara et al., 2023), the precise transfer mechanism remains incompletely elucidated. Notably, the detection of STARD3 within confined regions between LE/Lys and mitochondria suggests a potential mechanism for interaction associated with MCS formation (Zhang et al., 2002; Charman et al., 2010)

Structurally, STARD3 comprises the MENTAL domain (MLN64 NH2-terminal) at the N-terminus and the C-terminal START domain. The MENTAL domain anchors STARD3 to the endolysosomal membrane and contains a nonconventional FFAT motif crucial for interactions with vesicle-associated membrane proteins and the formation of MCS (Di Mattia et al., 2020). The START domain in the cytosol facilitates cholesterol positioning and mobilization between LE and other subcellular structures (Tsujishita and Hurley, 2000; Zhang et al., 2002; Charman et al., 2010; Alpy et al., 2013; van der Kant et al., 2013; Wilhelm et al., 2017; Nara et al., 2023).

STARD3 was identified as a lutein-binding protein because of its high homology with the carotenoid-binding protein (CBP) found in silkworms (Li et al., 2011). Binding studies demonstrated that STARD3 binds free lutein with high affinity (KD = 0.45 μM) and specificity; even so far, no other protein has been described to bind lutein with this affinity (Li et al., 2011; Horvath et al., 2016). The lutein-binding function of STARD3 resides within the C-terminal START domain as a tunnel-like hydrophobic cavity (Horvath et al., 2016), which can also accommodate a single cholesterol molecule with 1.5 mM affinity. Recently, Hempelmann et al., 2023 elegantly demonstrated the mobilization of sphingosine by STARD3 at inter-organellar level. Therefore, lutein cholesterol and maybe sphingosine share the same protein binding site, with lutein exhibiting a higher affinity. While STARD3 is primarily described as a lysosomal protein, evidence suggests its presence in the plasma membrane. Studies show STARD3 localization at the plasma membrane, with positive vesicles mediating cholesterol recycling (van der Kant et al., 2013) and the transfer of cholesterol from the endoplasmic reticulum to LE affecting the plasma membrane cholesterol pool (Wilhelm et al., 2017).

In this context, it is reasonable to speculate that STARD3 might prefer specific ligands based on their subcellular localization and the concentrations of available ligands. For example, under elevated lutein concentrations, the affinity of STARD3 for lutein may be heightened compared to cholesterol binding. This scenario could mitigate the adverse effects associated with STARD3-mediated cholesterol transportation to the mitochondria, which include its involvement in tumor progression and its correlation with increased mitochondrial cholesterol levels, thereby inhibiting apoptosis and increasing ROS (Asif et al., 2021). Our studies found that overexpression of STARD3 in mice livers resulted in hepatic damage and apoptosis (Tichauer et al., 2007). Moreover, overexpression of STARD3 in hepatocytes increased mitochondrial dysfunction by reducing membrane potential and increasing mitochondrial ROS levels, correlated with elevated mitochondrial cholesterol content (Balboa et al., 2017). Interestingly, this mechanism could explain the antiapoptotic and antioxidant effect of lutein, which likely occurs by lutein occupying the binding site of STARD3, thereby inhibiting its binding to cholesterol.

As was discussed before, STARD3 would not only reside in endosomes but can also cycle between the endocytic pathway and the plasma membrane (Alpy et al., 2001; Zhang et al., 2002; van der Kant et al., 2013; Vassilev et al., 2015). Therefore, considering this possibility, STARD3, located in the plasma membrane, could contribute to the lutein entry in the hepatocyte. Nevertheless, our prior findings have indicated that STARD3 is predominantly situated at the intracellular level (Balboa et al., 2017). However, under pathological conditions, such as steatosis, the subcellular localization of STARD3 remains understudied.

Regarding STARD3 gene expression, it has been described that its promoter region contains putative binding sites for lipid-responsive transcription factors, such as PPARs, retinoid X receptors (RXR), and SREBPs (Borthwick et al., 2009). Moreover, there is evidence that STARD3 expression is sterol-dependent in human THP-1 macrophages in which the increase in sterol content induces a decrease in STARD3 gene expression, while sterol depletion using methyl beta-cyclodextrin enhances STARD3 gene expression (Borthwick et al., 2009). In this context, it is interesting that lutein favors an increase in LDLR expression (also regulated by SREBP2) and activates PPARα in liver tissue in male Wistar rats (Soundarya et al., 2018), suggesting that lutein could regulate STARD3 expression. In line with this, STARD3 protein levels are correlated with lutein concentrations in the human brain (Tanprasertsuk et al., 2016). On the other hand, in insulinoma cells, it has been observed that the expression of STARD3 is not regulated by lutein (Pinto and Graham, 2016). However, this observation lacks clarity regarding the methodology employed for lutein administration, such as direct addition or incorporation via liposomes, as well as the duration of treatment. Therefore, we cannot guarantee proper cellular uptake of lutein or sufficient time for its intended effects to manifest.

Since in liver diseases such as MASLD, there is lipid accumulation, it is possible to infer that STARD3 levels will decrease, as in obesity models (Soffientini et al., 2014b).

Therefore, considering the hepatoprotective effects of lutein and the potential role of STARD3 as a lutein transporter, it is reasonable to propose that a decline in STARD3 expression could compromise liver health by restricting lutein uptake into cells. Additionally, lutein treatment may potentially restore physiological levels of STARD3 expression, thereby promoting liver health.

Considering the evidence that in hepatocytes, the overexpression of STARD3 facilitates the lipidation of exogenous apoA-I and promotes *de novo* biosynthetic pathways for neutral lipids, leading to an increase in triacylglycerol accumulation (Soffientini et al., 2014a) and that overexpression of STARD3 resulted in enhanced cholesterol efflux and reduced intracellular oxidized LDL accumulation (Almarhoun et al., 2021), it can be inferred that STARD3 plays a dual role in lipid metabolism depending on the cell type and this dual role could be determined by the binding to its ligands.

The role of STARD3 in the presence of lutein in hepatocytes is unclear. In this context, it remains unclear whether lutein competes against cholesterol for STARD3 binding, reducing TG accumulation, or if lutein has a hepatoprotective role independent of its interaction with STARD3. In this review, we have presented evidence of the hepatoprotective effect of lutein and proposed a mechanism for how lutein may exert this effect. The significance of its transporter, STARD3, is not well-documented, and we believe that further research into the role of STARD3 in the pathogenesis of hepatic steatosis is warranted. We posit that lutein’s effect may depend on its transporter, STARD3, making this protein crucial for the intracellular impact of lutein.

Our proposed model (Figure 2) positions lutein as a promising therapeutic candidate for hepatic steatosis.

## 5 Conclusion

Hepatic steatosis arises from an imbalance in various lipid homeostasis processes. Among these, we firmly believe that decreased lipophagy plays a crucial role in the development of steatosis, leading to reduced LD hydrolysis and availability of FA for critical reactions such as beta-oxidation. In this context, lutein, a known hepatoprotective agent, is proposed to exert its effect on the liver by enhancing lipophagy, and TFEB likely mediates this action. Additionally, we propose a model wherein STARD3 participates in the intracellular transport of lutein into hepatocytes, further emphasizing the relevance of this protein in the effects of the carotenoid. The data and evidence reviewed in this article strongly support our proposed model. While we recognize the need for additional *in vivo* models to validate our hypothesis, we believe that models currently under development, such as liver organoids derived from patients, could help address questions regarding the expression levels of STARD3 in MASLD patients *versus* controls, whether patients exhibit a different response to lutein compared to controls, and whether differences in TFEB expression, previously documented in MASLD patients, could also influence the response to lutein treatment. Based on the findings presented in this review, the current evidence supports lutein and STARD3 as promising nutraceutical and therapeutic targets, respectively, for treating hepatic steatosis.

## Footnotes

1
https://clinicaltrials.gov/study/NCT00596024
