# Correction: Exploring the lutein therapeutic potential in steatotic liver disease: mechanistic insights and future directions

**DOI:** 10.3389/fphar.2025.1633575

**Published:** 2025-06-25

**Authors:** Elisa Balboa, Faride Saud, Claudia Parra-Ruiz, Marjorie de la Fuente, Glauben Landskron, Silvana Zanlungo

**Affiliations:** ^1^ Center for Biomedical Research, Universidad Finis Terrae, Santiago, Chile; ^2^ Department of Gastroenterology, Faculty of Medicine, Pontificia Universidad Católica de Chile, Santiago, Chile

**Keywords:** hepatic steatosis, lipophagy, lutein, TFEB, StARD3, lipid droplet

In the published article, there was an error in Figure 2 as published. In the section of the figure labeled as “Step 2,” where STARD3 is shown at the plasma membrane, it is depicted with its N-terminal and C-terminal domains oriented toward the extracellular space. However, both the N- and C-terminal domains of STARD3 must be cytoplasmic. The corrected Figure 2 and its caption appear below.

**FIGURE 2:**
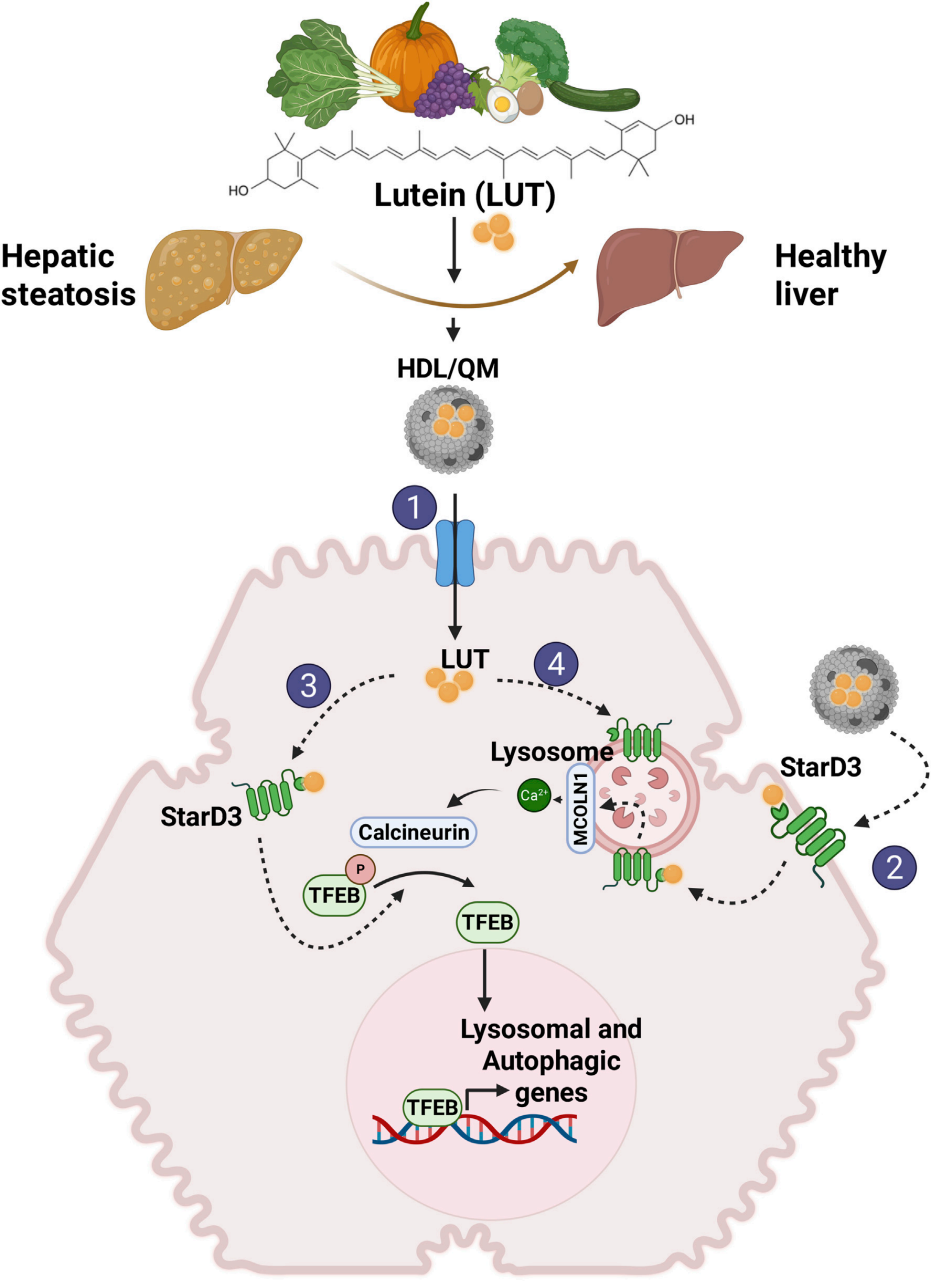
Proposed mechanisms involved in lutein-mediated hepatoprotective effects. We propose that independent of its antioxidant and antiapoptotic activity, lutein stimulates LD autophagy in the liver, reducing their accumulation and improving steatosis. This action is mediated through the activation of TFEB. Given lutein’s hydrophobic nature as a carotenoid, an intracellular transporter is required to exert its activity within the cell. We propose that STARD3 serves as this transporter. 1) To enter the cell, lutein in lipoproteins, primarily QM and HDL, binds to their transporters on the hepatocyte membrane (SRBI, LDLR, LRP1). 2) Additionally, lutein may directly enter the cell by binding to STARD3 on the plasma membrane. 3) Once inside the cell, lutein bound to STARD3 would directly or indirectly interact with TFEB, mediating its activation. 4) Another pathway for lutein entry involves endosomal uptake, where it binds to STARD3 in the lysosome, activating TFEB via the MCOLN1/Ca^2+^/calcineurina pathway.

The original version of this article has been updated.

